# Peer Review of “Representing Physician Suicide Claims as Nanopublications: Proof-of-Concept Study Creating Claim Networks”

**DOI:** 10.2196/39889

**Published:** 2022-07-01

**Authors:** Ariel Soares Teles

**Affiliations:** 1 Federal Institute of Maranhão Araioses Brazil

**Keywords:** physician suicide, suicide, suicide prevention, physician well-being, physician mental health, nanopublication, physician, doctor, mental health, semantic publishing, bibliometrics, claim network, information distortion, misinformation

*This is a peer-review report submitted for the paper “Representing Physician Suicide Claims as Nanopublications: Proof-of-Concept Study Creating Claim Networks.”*

## Round 1 Review

This paper [1] presents a study focused on “the use of nanopublications as a scientific publishing approach to establish a citation network of claims drawn from a variety of media concerning the rate of suicide of US physicians.” The study finds interesting results, and I have the following comments and concerns.

### Specific Comments

#### Major Comments

1. Consider the sentence “To our knowledge, no such application to this field has previously been done.” Authors should provide related work to argue this. Comparison with previous works is missing in the paper. Are there others related to nanopublications?

2. The paper would improve if examples (at least one) of nanopublications used in the data source were added. This would be illustrative.

3. Reference Leung et al [2] 2019 has been published and is apparently peer-reviewed. Check if there are other references to be added in the data source.

4. Consider these two sentences: “A subset of articles from the literature search were identified that made an assertion (claim) about the annual rate of US physicians who die of suicide. Additional articles published between August 2019 and March 2020 have been identified and manually added to the article set used for this study.” I think these sentences should be unpacked. How were these two steps performed?

5. The main results of this paper are in Table 1, which “revealed that (1) the network is not fully connected, (2) no single primary source of the claim could be identified, and (3) all end-point citations either had a claim with no further citation, had no apparent claim, or could not be accessed to verify the claim.” This is interesting, but what was the rationale for using nanopublications as a tool in the methodology? Could these results be found using snowballing as a review method?

6. What are the contributions of the paper? They could be explicitly declared. Moreover, the objective of the paper should be better declared—“In this paper, we aim to create nanopublications from assertions relating to physician suicide incidence.” I think this is not the same from the abstract, which is much better.

#### Minor Comments

7. eg to eg, (add comma)

8. et al to et al. (add dot)

9. Figure 1 is in low quality.

10. Remove “-” from URLs:

http://purl.org/np/RAqWlNPJt3Eb4HkmPCpjaiR “-”HGCzKIZag6cBNMkG8nxu6I

## Round 2 Review

I congratulate the authors for their work. All my questions were answered, and concerns addressed. Thank you!

## References

[1] Leung TI, Kuhn T, Dumontier M (2022). Representing Physician Suicide Claims as Nanopublications: Proof-of-Concept Study Creating Claim Networks. JMIRx Med.

[2] Leung TI, Snyder R, Pendharkar Ss, Chen CA (2020). Physician suicide: a scoping literature review to highlight opportunities for prevention. GPA.

